# Discovery of Novel Chinese Medicine Compounds Targeting 3CL Protease by Virtual Screening and Molecular Dynamics Simulation

**DOI:** 10.3390/molecules28030937

**Published:** 2023-01-17

**Authors:** Jin Cheng, Yixuan Hao, Qin Shi, Guanyu Hou, Yanan Wang, Yong Wang, Wen Xiao, Joseph Othman, Junnan Qi, Yuanqiang Wang, Yan Chen, Guanghua Yu

**Affiliations:** 1 School of Pharmacy, Jiangsu Vocational College of Medicine, Yancheng 224005, China; 2 Department of Pharmaceutical Sciences and Computational Chemical Genomics Screening Center, School of Pharmacy, National Center of Excellence for Computational Drug Abuse Research, University of Pittsburgh, Pittsburgh, PA 15261, USA; 3 School of Pharmacy and Bioengineering, Chongqing University of Technology, Chongqing 400054, China; 4 College of Pharmacology Sciences, Zhejiang University of Technology, Hangzhou 310014, China

**Keywords:** SARS-CoV-2, 3CL protease, virtual screening, traditional Chinese medicine, molecular dynamics simulations

## Abstract

The transmission and infectivity of COVID-19 have caused a pandemic that has lasted for several years. This is due to the constantly changing variants and subvariants that have evolved rapidly from SARS-CoV-2. To discover drugs with therapeutic potential for COVID-19, we focused on the 3CL protease (3CL^pro^) of SARS-CoV-2, which has been proven to be an important target for COVID-19 infection. Computational prediction techniques are quick and accurate enough to facilitate the discovery of drugs against the 3CL^pro^ of SARS-CoV-2. In this paper, we used both ligand-based virtual screening and structure-based virtual screening to screen the traditional Chinese medicine small molecules that have the potential to target the 3CL^pro^ of SARS-CoV-2. MD simulations were used to confirm these results for future in vitro testing. MCCS was then used to calculate the normalized free energy of each ligand and the residue energy contribution. As a result, we found ZINC15676170, ZINC09033700, and ZINC12530139 to be the most promising antiviral therapies against the 3CL^pro^ of SARS-CoV-2.

## 1. Introduction

The ongoing COVID-19 worldwide pandemic (declared by the WHO) has caused more than 6.58 million deaths and 632.9 million cases worldwide as of 24 October 2022 [1]. Even though the symptoms are relatively mild, the high transmission rate has caused a crisis that has resulted in a variety of supply chain and economic issues as workplaces and ports globally have shut down [2,3]. Treatments to fight the virus are often thwarted by the virus’s ability to mutate and evolve. Severe acute respiratory syndrome coronavirus 2 (SARS-CoV-2) has already mutated into several different variants such as Alpha, Beta, Gamma, Delta, Epsilon, Zeta, Iota, Theta, and Kappa [4]. The newer variants and subvariants are not only more transmissible but also become more difficult for host antibodies to recognize. This deadly combination combats the long-term immunity that vaccines are trying to achieve.

Therefore, the innovations of less time-consuming antiviral drug development methods such as drug repurposing are needed to keep up with the rates of infection to eventually solve the problem [5]. Drug repurposing is one of the quickest ways to develop effective therapeutics against COVID-19 [6]. For example, in one study, researchers conducted experiments to test broad-spectrum antivirals (ribavirin, interferons, and cyclophilin inhibitors) against coronaviruses [7]. However, since the drug repurposing technique utilizes a “broad spectrum” treatment strategy, it may be too broad and likewise not very effective. This study also had a limitation in that it only screened a small range of drug varieties. Thus, we collected 225,667 ligand compounds from the traditional Chinese medicine (TCM) database [8] to offer a more comprehensive study with a higher scope of drug variety. One of the common processes of drug repurposing is to use both ligand-based virtual screening and structure-based virtual screening, which is an approach that utilizes the Schrödinger Maestro screening process to view the docking scores of the ligand binding process. However, focusing only on the docking scores without taking key residues into account may increase the false positive rate [9]. Therefore, all these points need to be considered and overcome when creating a new treatment process for SARS-CoV-2.

SARS-CoV-2 is an enveloped virus that belongs to the beta coronavirus family with a complex, single-stranded RNA genome (~30 kb) similar to the other known human coronaviruses, CoVs (SARS-CoV, MERS-CoV, HKU1, NL63, OC43, and 229E) [10]. SARS-CoV-2 contains spike proteins, membrane proteins, envelope proteins, and nucleocapsid proteins. SARS-CoV-2 enters the host’s cells through receptor binding with its viral spike proteins and membrane fusion, which allows the virus to release its RNA that causes the cell to replicate and produce several subgenomic RNAs [11]. The SARS-CoV genome consists of two open reading frames that can encode two polyproteins, pp1a and pp1ab [12]. Pp1a and pp1ab can moderate the mechanism of viral replication and transcription [13]. Genome analysis has shown that SARS-CoV-2 is similar to bat CoV, which has evolved to bind to human ACE2 receptor proteins with higher affinity than other coronaviruses [14]. Using several antiviral strategies, researchers are creating new ways to target and inhibit the binding of the spike protein with the receptor proteins. Therefore, the solutions based on targeting coronavirus rely on blocking the virus binding to receptors, inhibiting virus replication, preventing the synthesis of viral RNA, and inhibiting the virus self-assembly process [15,16,17]. The 3CL main protease protein is an effective target because of its crucial role in viral maturation and post-translational processing of replicase polyproteins [18,19].

Recently, the use of computational methods has become increasingly popular because of their high efficiency and effectiveness [20]. Applying computational methods in drug discovery and development can also save time, costs, and manpower [13]. Since 3CL^pro^ is the most suitable target for new drug therapeutics, the most efficient way to discover a treatment plan is to utilize structural bioinformatics strategies to determine the best ligand structures to bind to it (3CL^pro^). In the present work, we conducted both ligand-based virtual screening and structure-based virtual screening to screen the Chinese medicine small molecule that has the potential to target the 3CL^pro^ of SARS-CoV-2. MD simulations were then used to confirm these results for future in vitro testing. Furthermore, we used MCCS screening methods [9] to search for the ligands that had the most and strongest bonds to the key residues in 3CL^pro^. The ligands with the strongest bonds with key residues would most likely be used in effective antiviral therapeutic treatments to combat SARS-CoV-2. All these components (docking score, MCCS normalized total free energy, and MD binding free energy) were used to determine the most suitable ligands to combat SARS-CoV-2.

## 2. Results

### 2.1. Virtual Screening of TCM

We performed ligand-based virtual screening of the traditional Chinese medicines database (TCM) to exclude ligands that do not have the potential to bind with 3CL^pro^ within the binding pocket of the five inhibitors listed above. We narrowed down the 177,764 TCM compounds to 17,939 compounds that have similar pharmacophore features to the five inhibitors listed above. Then we conducted structure-based virtual screening for these 17,939 compounds and listed the top 10 ligands with the best docking scores. The suitable ligands found in this study are ZINC15676170, ZINC15675325, ZINC12529667, ZINC13550544, etc. (Table 1). The best docking score, −9.178 kcal/mol, was reported for ZINC15676170. The candidates shown in Table 2 have high potential against the 3CL^pro^ of SARS-CoV-2.

### 2.2. Molecular Dynamics Simulation of the Top Ligands

To better understand the conformational and dynamics features of the top ligands against the 3CL^pro^ of SARS-CoV-2, molecular dynamics simulations were performed for the top 10 ligands. Dynamics features of the top 10 ligands were calculated after a 100 ns MD simulation.

As we can see from Table 2, the binding free energies predicted by the molecular dynamics simulation of ZINC09033700, ZINC15676170, and ZINC12530139 are −6.8953 kcal/mol, −4.3351 kcal/mol, and −3.0082 kcal/mol, respectively, which indicates that the binding of 3CL^pro^ with ZINC09033700, ZINC15676170, and ZINC12530139 is stable.

### 2.3. The Time Course of Root-Mean-Square Deviations (RMSD) Analysis

The stabilities of all the top 10 systems were evaluated by using the time course of root-mean-square deviations (RMSD) analysis. As shown in Figures S1 and S2, the results indicated that the stabilities of all ten systems were high with acceptable deviation. The average RMSD for all systems ranged between 1 and 5Å.

### 2.4. The Root-Mean-Square Fluctuation (RMSF) Analysis

**Figure 1:**
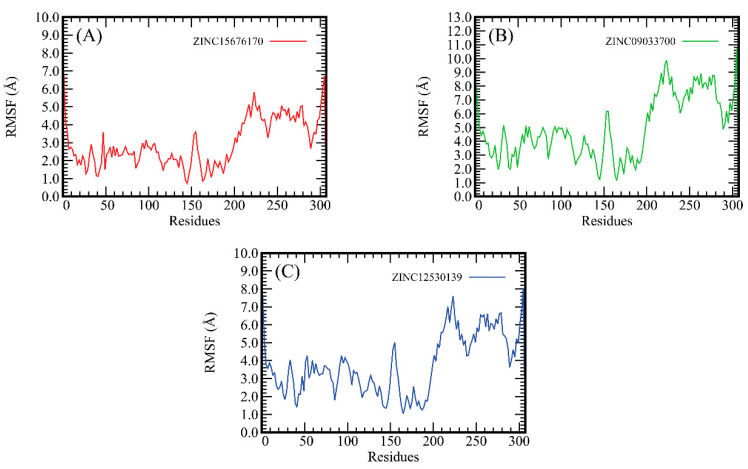
RMSFs of all systems including ZINC15676170 (red), ZINC09033700 (green), and ZINC12530139 (blue). The abscissa represents the residue number. The ordinate represents the value of Root-Mean-Square Fluctuation, and the unit is angstrom.

The RMSF delineated the average deviation of a particle over time from a reference location. As shown in Figure 1, the deviation pattern of these three systems is very similar. Significant fluctuations were observed within the regions 0–10, 40–50, 85–95, 145–160, 240–250, and 290–307, which represent the loop regions. The regions 10–20, 50–90, 100–110, and 250–275 are very stable, which represent helix strands or β turns. These findings are consistent with the structure of the 3CLpro of SARS-CoV-2. The region 200–220 fluctuates greatly, which may represent a connecting loop. Fluctuations in other regions were relatively flat. These findings indicate that the 3CLpro of SARS-CoV-2 is stabilized by the binding of ZINC15676170, ZINC09033700, and ZINC12530139.

### 2.5. Energy Decomposition of Three Potential Inhibitors and Their Complex by MM/GBSA Calculation and MCCS

Residue energy contribution is an important factor to be considered during the screening process. By acknowledging the importance of the bonds in key residues, the results from the screening process are more accurate and effective. The residue energy contributions shown in Table 3, Table 4 and Table 5 were predicted by MM/GBSA calculation. We only listed the top 10 residues. MCCS utilized the jdock function to calculate and display the energy contributions of each residue. To identify residues classified as “key residues”, we only chose residues that had an inter-ligand free energy contribution of <−0.3 kcal/mol (the lower values represent the stronger binding between the ligand and the receptor). The values differed from the docking scores because MCCS calculates total free energy with the jdock function, while Glide, the ligand docking program we used on Maestro, calculates docking scores with the SP and XP GlideScore functions.

**Figure 2:**
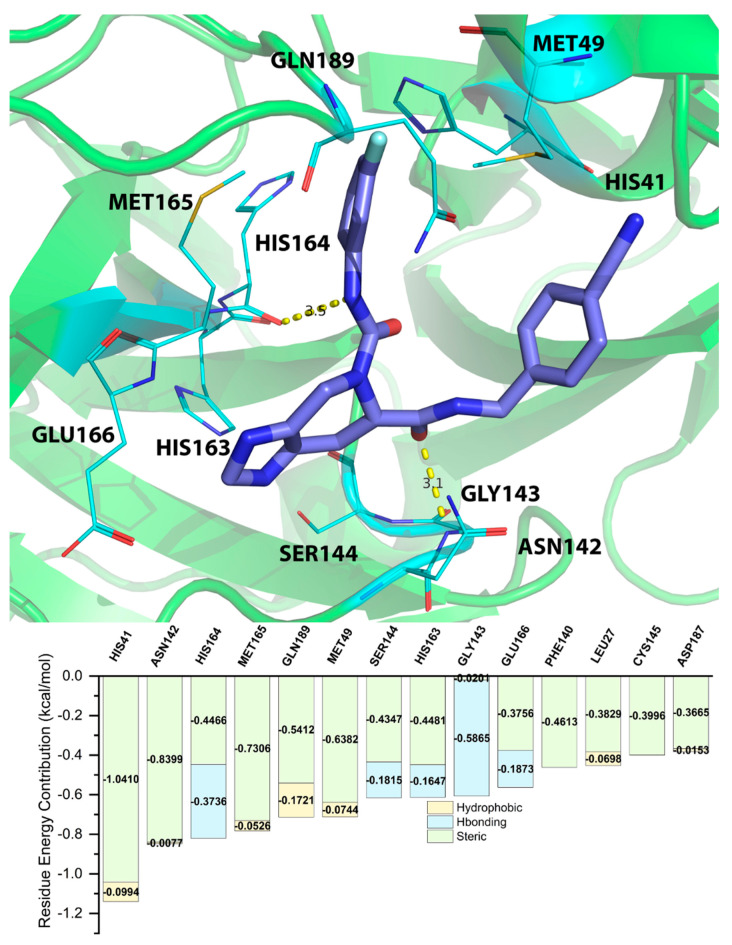
Binding poses between ZINC15676170 and 3CL^pro^ of SARS-CoV-2 predicted by MD simulation and residue energy contribution between ZINC15676170 and 3CL^pro^ of SARS-CoV-2 predicted by MCCS.

The residue energy contribution between ZINC15676170 and the 3CL^pro^ of SARS-CoV-2 was analyzed by both MD simulation and MCCS. As shown in Figure 2, the binding pose between ZINC15676170 and the 3CL^pro^ of SARS-CoV-2 predicted by MD simulation indicated that the nitrogen atom of ZINC15676170 can form a hydrogen bond with the oxygen atom of HIS164, with the distance of 3.5Å, and the oxygen atom of ZINC15676170 can form a hydrogen bond with the nitrogen atom of GLY143, with the distance of 3.1Å. The MCCS prediction results indicated that the HIS164 and GLY143 have a very high chance of forming a hydrogen bond with ZINC15676170, which is consistent with the MD simulation prediction.

**Figure 3:**
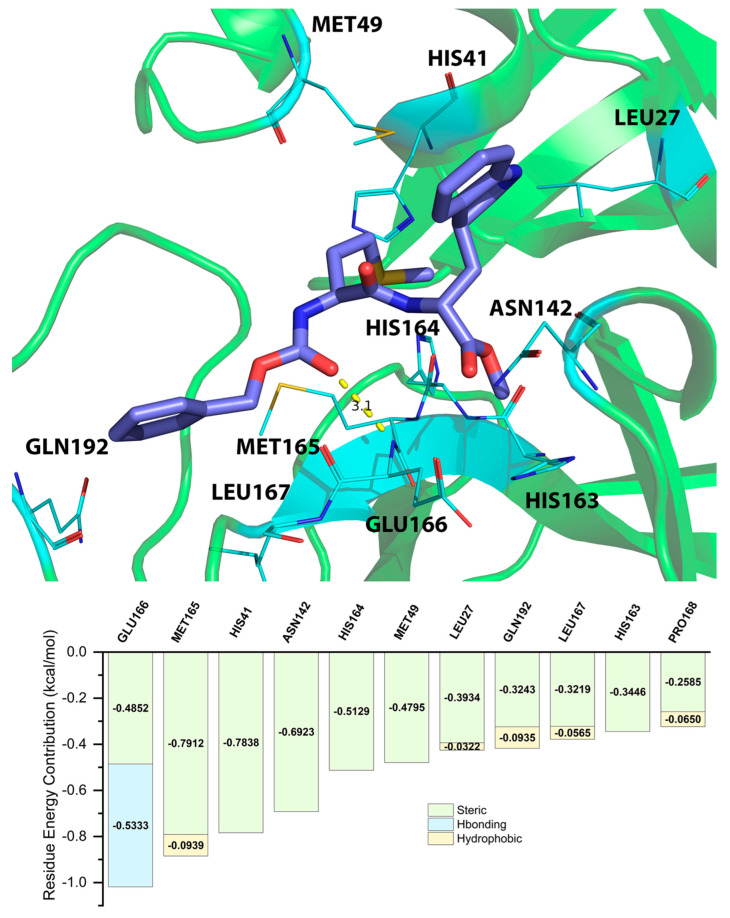
Binding poses between ZINC09033700 and 3CL^pro^ of SARS-CoV-2 predicted by MD simulation and residue energy contribution between ZINC09033700 and 3CL^pro^ of SARS-CoV-2 predicted by MCCS.

The residue energy contribution between ZINC09033700 and the 3CL^pro^ of SARS-CoV-2 was analyzed by both MD simulation and MCCS. As shown in Figure 3, the binding pose between ZINC09033700 and the 3CL^pro^ of SARS-CoV-2 predicted by MD simulation indicated that the oxygen atom of ZINC15676170 can form a hydrogen bond with the nitrogen atom of GLU166, with the distance of 3.1Å. The MCCS prediction results indicated that the GLU166 has a very high chance of forming a hydrogen bond with ZINC09033700, which is consistent with the MD simulation prediction.

**Figure 4:**
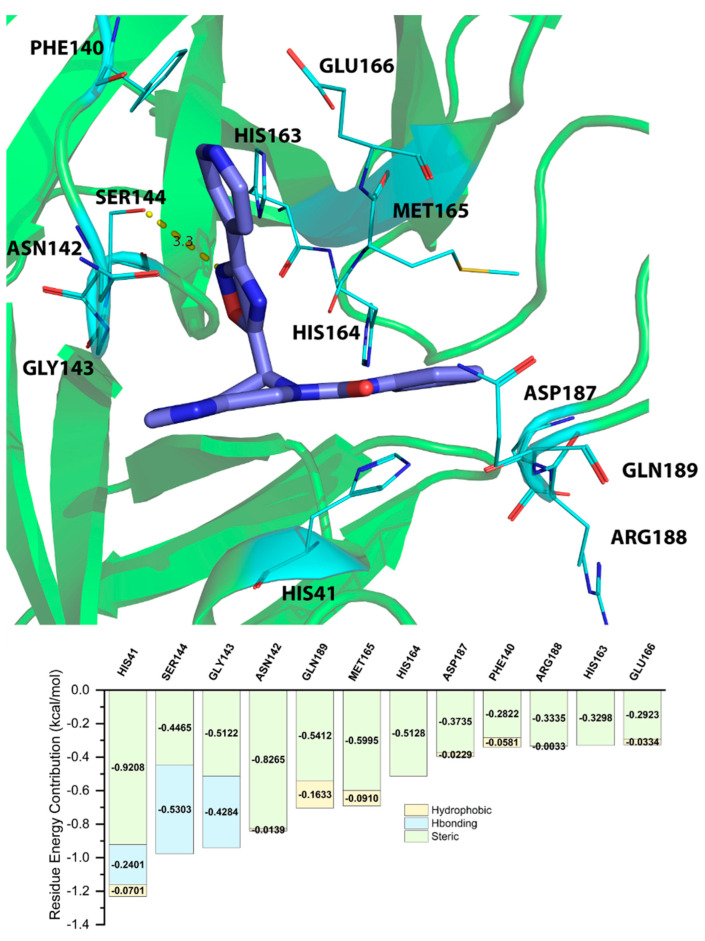
Binding poses between ZINC12530139 and the 3CL^pro^ of SARS-CoV-2 predicted by MD simulation and residue energy contribution between ZINC12530139 and 3CL^pro^ of SARS-CoV-2 predicted by MCCS.

The residue energy contribution between ZINC12530139 and the 3CL^pro^ of SARS-CoV-2 was analyzed by both MD simulation and MCCS. As shown in Figure 4, the binding pose between ZINC12530139 and the 3CL^pro^ of SARS-CoV-2 predicted by MD simulation indicated that the nitrogen atom of ZINC15676170 can form a hydrogen bond with the oxygen atom of SER144, with the distance of 3.3Å. The MCCS prediction results indicated that the SER144 has a very high chance of forming a hydrogen bond with ZINC12530139, which is consistent with the MD simulation prediction.

The purpose of MD simulation is to simulate a dynamic process, while MCCS uses the jdock function to predict a static binding pose. The representative conformation is the conformation found in 1000 snapshots of the MD simulation that is closest to the average conformation. The energy decomposition of MM/GBSA predicted by the MD simulation represents the energy decomposition of the representative conformation. Therefore, there may be some difference between the energy decomposition of MM/GBSA and the energy decomposition of MCCS. Although the rankings of residues were not identical due to different scoring mechanisms for residues, we found that at least 8 out of the top 10 residues (80%) were consistent. The accuracy of MCCS prediction has been approved by another manuscript [21].

From the energy contribution between the three potential inhibitors and 3CL^pro^ calculated by MM/GBSA and MCCS, we can predict that HIS41, ASN142, GLY143, SER144, HIS163, HIS164, MET165, GLU166, and GLN189 are the key residues that play an important role within the binding between the potential inhibitors and 3CL^pro^. Among the key residues, GLY143, SER144, HIS164, and GLU166 have a very high potential to form hydrogen bonds with potential inhibitors. Steric hindrance and hydrogen bonding are the main forms of energy contribution. As shown in Figure S3, we also analyzed the hydrogen-bonding occupancy of ZINC15676170, ZINC09033700, and ZINC12530139. In ZINC15676170, the hydrogen bonds of GLY143 and GLU166 accounted for 25.98% and 15.6% of the trajectory, respectively. In ZINC09033700, the hydrogen bonds of GLU166 accounted for 21.5% of the trajectory. In ZINC12530139, the hydrogen bonds of GLY143 and SER144 accounted for 16.5% and 0.4% of the trajectory, respectively.

## 3. Methods

### 3.1. Protein and Ligand Structure Organization

In this study, we retrieved the three-dimensional coordinates of 3CL^pro^ from RCSB protein data bank (PDB id: 6LU7) [22]. This structure consists of N3 co-crystal inhibitor and 3CL^pro^. Protein preparation wizard implemented in Schrödinger software (Schrödinger, LLC, New York, NY, USA) was used to prepare and optimize the structure of 6LU7, which included filling in missing side chains, using Prime and adding hydrogens. The OPLS3 force field was used for protein-energy minimization. LigPrep was used to prepare the ligands.

### 3.2. TCM Database

The chemical compounds used in this study were collected from the TCM database. We chose to use the TCM database because the TCM database is currently the largest and most comprehensive database on traditional Chinese medicine [8]. We collected 225,667 natural compounds, which were then filtered through using Lipinski’s rule of five (we excluded any natural compounds that violated this rule). This narrowed 225,667 natural compounds down to 177,764 ligands, which were more likely to be a hit. These ligands were then uploaded to Maestro for scoring function calculations and virtual screening.

### 3.3. Inhibitor Filtering by Ligand-Based Pharmacophore Model

In this study, we collected 5 inhibitors that bonded well to 3CL^pro^ in SARS-CoV-2. These include the N3, GRL-2420, ML188, CALPAIN1, and nirmatrelvir inhibitors retrieved from Protein databank 6LU7, 7JKV, 7L0D, 7LBN, and 7U29 respectively. Among these inhibitors, we conducted the ligand-based virtual screening process, which uses structural commonalities between the 5 inhibitors to accurately identify the key pharmacophore model that plays an important role in the binding process. The pharmacophores that were inserted into the crystal structure of 3CL^pro^ let us identify the key residues and the interaction type that play a major role in the binding between the inhibitor and 3CL^pro^ of SARS-CoV-2. Each inhibitor contains 4 or 5 pharmacophore features that can form interactions with 3CL^pro^, and we collected the ligands that contain at least 2/4 or 3/5 pharmacophore features as the inhibitors listed above. This allowed us to narrow down the 177,764 TCM compounds to 17,939 compounds that have similar pharmacophore features as the 5 inhibitors listed above.

### 3.4. Virtual Screening and Selection Method

The Maestro software allows us to screen and select ligands from the TCM database. First, we used the Maestro virtual screening software to display the preprocessed and minimized structures of the 6LU7 protein. Then, we used several visual tools to find the various bonds (hydrogen bonds, salt bridges, and π-π stacks) between the ligand and the crystal structure of 3CL^pro^. The bonds between the key residues of 3CL^pro^ and the ligand are considered very important because of the lower binding free energy between the key residues of 3CL^pro^ and the ligands, thus the more stable the complex formed by ligand and 3CL^pro^ is. The set of 17,939 ligands were docked into the active site of 3CL^pro^ and then displayed in the order of highest (most negative) docking score. The docking score was calculated using the Glide program that utilized the SP and XP GlideScore scoring functions. We selected the top 10 ligands (according to docking score). The Schrödinger Maestro tools calculate various bond types (hydrogen bonds, salt bridges, and π-π stacks), which help us screen the number of bonds that each ligand has with the protein.

### 3.5. Molecular Dynamics (MD) Simulation

We performed molecular dynamics simulations on the top 10 ligands and 3CL^pro^ complexes to further validate our virtual screening results. The MD simulations were carried out in a cubic box (~90Å × 90Å × 90Å) with periodic boundary conditions to model a representative part of the interface devoid of any arbitrary boundary effects [23]. The system was solvated in a 0.15 mol/L NaCl solution, including 15,021 water molecules, 40 Cl^−^ and 44 Na^+^ ions. The systems were protonated prior to the MD simulations. Special caution was applied to histidine residues, which can be ionized at pH 7.40. AMBER ff14SB force field [24] and GAFF [25] were applied to the protein and ligands, respectively. The TIP3P water model was used to treat the water molecules [26]. Subsequently, the PMEMD.mpi and PMEMD.cuda modules in the AMBER16 software package were used to perform the MD simulations [27,28,29].

Before the MD simulations, we first relaxed the complex by minimizing 5000 cycles to avoid the possible spatial conflicts—500 cycles of steepest descent and 4500 cycles of conjugated gradient minimization [30]. After minimization, it entered the heating phase. In this step, each system was gradually heated from 0 K to 303.15 K, followed by equilibrium and production stages (303.15 K) with a time step of 2 fs. Periodic boundary conditions were used to maintain constant temperature and pressure (NPT) ensembles. The pressure was set to 1 atm and was controlled by an anisotropic (x-, y-, z-) pressure scaling protocol, and the pressure relaxation time was 1 ps. Langevin dynamics were used to control the temperature to keep the temperature at the collision frequency of 2 ps^−1^ [31,32]. The particle grid Ewald (PME) method [33,34] was used to deal with long-distance static electricity, and the critical value of actual spatial interaction was set to 10 Å. The SHAKE algorithm was used to constrain all covalent bonds related to hydrogen atoms [35]. Both systems were subjected to 100 ns MD simulations, and a trajectory of the simulated system was saved every 100 ps.

### 3.6. Molecular Mechanics/Generalized Born Surface Area (MM/GBSA) Calculation

In MM/GBSA, their binding free energy (ΔG_bind_) of 3CL/molecule was calculated by the following equation:

ΔG_bind_ = ΔH − TΔS ≈ ΔE_MM_ + ΔG_sol_ − TΔS

Among them, ΔE_MM_ was the gas phase MM energy during binding, ΔG_sol_ and −TΔS were the changes in solvation free energy and the conformational entropy on ligand binding, respectively [30]. T was the absolute temperature and ΔS was the molecule’s entropy [36]. ΔE_internal_ specifically refers to bond, angle, and dihedral energy, which together with electrostatic ΔE_electrostatic_ energy and van der Waals force ΔE_vdw_ form ΔE_MM_ [30]. The specific calculation formula is as follows:

ΔE_MM_ = ΔE_internal_ + ΔE_electrostatic_ + ΔE_vdw_

The energy contribution of ΔG_sol_ includes electrostatic solvation energy (polar contribution) ΔG_GB_ and non-electrostatic solvation component ΔG_SA_ (non-polar contribution). The non-polar contribution obtained by fitting the solvent accessible surface area (SASA) [37] and linear combination of pairwise overlaps (LCPO) model [38,39]. The specific calculation formula is as follows:

ΔGsol = ΔGGB + ΔGSA

In addition, the energy of each residue could also be calculated. Energy analysis could analyze the energy contribution of key residues in the decomposition of the binding process [40].

### 3.7. Molecular Complex Characterizing System

As stated in the introduction before, looking only at the docking scores produces false positive results [9]. Considering the key residues in the docking process can make the results of virtual screening more convincing and make it easier for biological experiments to verify the inhibitory efficacy of ligands against 3CL^pro^. The energy contribution of key residues was calculated by Molecular Complex Characterizing System (MCCS). Firstly, Chimera (version 1.15) [41] was applied to fix the incomplete side chain of protein PDBs. To be more specific, the full protein structures were scanned by Chimera and then the incomplete residues were revealed. The truncated side chains were replaced with entire side chains of the same residue type with the aid of the Dunbrack rotamer library [42]. Secondly, the polar hydrogens, Vina force field, and Gasteiger charges were added by VEGA [43]. Thirdly, the PDB format was converted to the PDBQT format. For ligand files, VEGA was first used to prepare the ligand PDBs as the protein preparation. Secondly, PROPKA (version 3.1) [44] was used to predict the pKa values of ligands [45]. MCCS would protonate the tertiary (3°) amide in the compounds when the computed pKa value of the ligands was greater than or equal to the supplied pH (7.4 by default). Thirdly, the torsions of ligands were determined by VEGA, and the ligand PDB format was converted to the PDBQT format. Finally, the PDBQT files of the ligand and the protein and the pKa file of the ligand were used as inputs into MCCS, which includes scoring and docking with jdock to calculate the residue energy contribution.

Jdock is the core implementation of the MCCS algorithm, which can generate nine different energy contribution vectors in total, including Gauss, Gauss1, Gauss2, hydrogen-bonding, hydrophobic, non-steric (hydrogen-bonding and hydrophobic), repulsion, steric (Gauss1, Gauss2, and repulsion), and total energy contribution [9]. The docking mechanism in jdock can generate up to 999 bound poses. The top 10-15 key residues were then recorded and used in the virtual screening process to filter out false positive results.

## 4. Conclusions

The SARS-CoV-2 virus relies on receptor binding using spike proteins to release its genetic material and spread the infection of the virus to other cells. Therefore, techniques that utilize drugs to inhibit the spike glycoprotein or 3CL^pro^ are effective treatments against SARS-CoV-2 [11]. In this study, the molecular docking and molecular dynamics simulations proved that ZINC15676170, ZINC09033700, and ZINC12530139 are very likely candidates for an antiviral therapeutic treatment process against SARS-CoV-2. MCCS displayed the energy contributions and key residues that greatly helped the virtual screening process, and MD simulations validated these results. In order to evaluate our virtual screening results, we again used Glide docking in Schrödinger to dock N3, GRL-2420, CALPAIN1, nirmatrelvir, and ML188 inhibitors retrieved from the protein databases 6LU7, 7JKV, 7LBN, 7U29, and 7L0D, respectively, into 3CL^pro^. The docking scores of N3, GRL-2420, CALPAIN1, nirmatrelvir, and ML188 are −10.073, −9.655, −7.28, −6.552, and −6.002 kcal/mol, respectively. N3 can form a covalent bond with 3CL^pro^, and GRL-2420 can also form a covalent bond with the Cys-145 of 3CL^pro^, which account for the high docking scores of N3 and GRL-2420 [46,47]. The docking scores of ZINC15676170, ZINC0903370, and ZINC12530139 are −9.178, −8.799, and −8.774, respectively, which are significantly higher than the docking scores of CALPAIN1, nirmatrelvir, and ML188. We also performed MD simulations of ZINC15676170, ZINC0903370, and ZINC12530139 with the 3CLpro of SARS-CoV-2, respectively, and the system was subjected to 500 ns MD simulations. The time course of the root-mean-square deviations (RMSD) plot (Figure S4) of the 500 ns MD simulations indicated the bindings of ZINC15676170, ZINC0903370, and ZINC12530139 with the 3CLpro of SARS-CoV-2, respectively, are stable. Obviously, we still need to conduct in vitro tests and human clinical studies to determine the efficacy of these drugs and to further validate our results. However, in silico computational prediction has proven to be a large asset for drug discovery and repurposing. The speed and efficiency of such studies have been essential to the development of effective antiviral therapeutic treatments [20].

## Figures and Tables

**Table 1:** Top 10 ligands with the best docking scores predicted by Glide.

Rank	ZINC ID	Structure	Docking Score (kcal/mol)	Molecular Weight (amu)	LogP Value
1	ZINC15676170	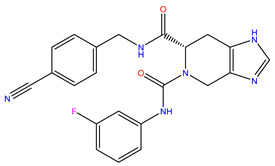	−9.178	418.432	3.228
2	ZINC15675325	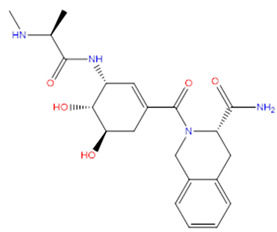	−9.171	417.486	−2.19
3	ZINC12529667	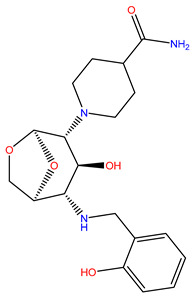	−9.124	378.449	−1.228
4	ZINC13550544	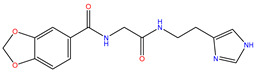	−9.031	317.325	−0.354
5	ZINC03838803	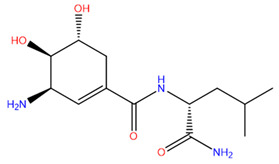	−8.947	286.352	−2.335
6	ZINC12664661	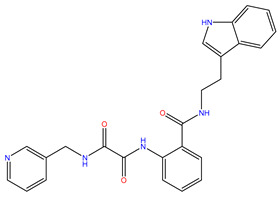	−8.845	442.499	2.476
7	ZINC09033700	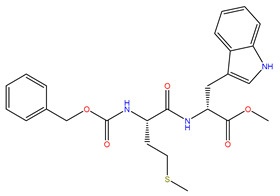	−8.799	483.589	3.683
8	ZINC12530139	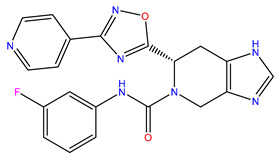	−8.774	407.409	2.525
9	ZINC00198624	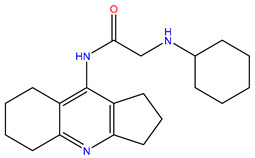	−8.704	329.488	1.703
10	ZINC08299537	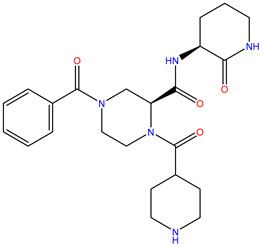	−8.602	442.54	−1.292

The table above shows the structure, docking score, molecular weight, and logP value of the ligands with the highest therapeutic potential against 3CL^pro^ of SARS-CoV-2.

**Table 2:** MD binding free energy ranking of top 10 ligands with the highest docking score.

Rank	ZINC ID	MD Binding Free Energy (kcal/mol)
1	ZINC09033700	−6.8953
2	ZINC15676170	−4.3351
3	ZINC12530139	−3.0082
4	ZINC15675325	−1.8083
5	ZINC12529667	−1.3637
6	ZINC00198624	−0.1468
7	ZINC12664661	0.0053
8	ZINC03838803	0.4986
9	ZINC08299537	0.6072
10	ZINC13550544	8.7553

**Table 3:** Energy decomposition of MM-GBSA free energy for ZINC15676170. All energies are in kcal/mol.

Residue	E_VDW_	E_EEL_	E_INT_	G_POL_	E_SAS_	E_MM_	E_EELPOL_	E_VDWEELPOL_	G_GBSA_	G_MM/GBSA_
CYS145	−1.8152	−1.767	0	0.0997	−1.2208	−3.5822	−1.6673	−3.4824	−1.121	−4.7032
ASN142	−2.2802	−0.8786	0	0.0751	−1.3824	−3.1588	−0.8035	−3.0838	−1.3073	−4.4662
MET165	−2.4885	0.1252	0	−0.396	−1.5208	−2.3633	−0.2709	−2.7594	−1.9169	−4.2802
HIS164	−2.066	−1.1163	0	0.3195	−0.9614	−3.1823	−0.7968	−2.8628	−0.6419	−3.8242
GLY143	−0.7966	−1.5772	0	−0.053	−0.6921	−2.3738	−1.63	−2.4266	−0.7448	−3.1187
HIS163	−0.9209	−1.5059	0	−0.055	−0.576	−2.4268	−1.5613	−2.4822	−0.6314	−3.0582
MET49	−1.3443	−0.2809	0	0.1433	−1.1549	−1.6252	−0.1376	−1.4819	−1.0116	−2.6368
GLU166	−0.8813	−2.6098	0	1.815	−0.877	−3.4911	−0.7948	−1.6761	0.938	−2.553
PHE140	−0.8967	−0.9905	0	0.4139	−0.4562	−1.8872	−0.5766	−1.4733	−0.0423	−1.9295
HIS41	−1.1417	0.2094	0	−0.212	−0.7811	−0.9322	−0.0026	−1.1442	−0.993	−1.9253

E_VDW_: van der Waals energy; E_EEL_: electrostatic energy; E_INT_: internal energy; G_POL_: the polar part of solvation free energy; E_SAS_: non-polar solvation component; E_MM_: gas phase Molecular Mechanics energy during binding; E_EELPOL_: electrostatic energy and the polar part of solvation free energy; E_VDWEELPOL_: van der Waals energy and electrostatic energy and the polar part of solvation free energy; G_GBSA_: Generalized Born Surface Area free energy; G_MM/GBSA_: Molecular Mechanics/Generalized Born Surface Area free energy.

**Table 4:** Energy decomposition of MM/GBSA free energy for the ZINC09033700. All energies are in kcal/mol.

Residue	E_VDW_	E_EEL_	E_INT_	G_POL_	E_SAS_	E_MM_	E_EELPOL_	E_VDWEELPOL_	G_GBSA_	G_MM/GBSA_
MET165	−3.6331	−2.335	0	0.827	−2.2815	−5.9681	−1.508	−5.1411	−1.4545	−7.4226
ASN142	−2.2616	−3.8489	0	1.0485	−1.8162	−6.1106	−2.8004	−5.062	−0.7676	−6.8782
HIS164	−2.005	−3.4553	0	0.29	−1.2107	−5.4603	−3.1653	−5.1703	−0.9207	−6.381
MET49	−2.363	−1.6125	0	0.8082	−1.6008	−3.9755	−0.8043	−3.1673	−0.7926	−4.7681
GLU166	−1.7504	2.5706	0	−3.6206	−1.093	0.8203	−1.0499	−2.8003	−4.7136	−3.8933
CYS145	−1.5458	−0.2229	0	−0.1515	−1.2428	−1.7687	−0.3743	−1.9201	−1.3943	−3.1629
HIS41	−1.3246	−0.288	0	−0.015	−1.01	−1.6126	−0.303	−1.6276	−1.025	−2.6376
LEU167	−1.2952	0.2722	0	−0.3641	−0.8727	−1.023	−0.0919	−1.3871	−1.2368	−2.2598
ASP187	−1.1167	−1.877	0	1.5806	−0.7113	−2.9937	−0.2963	−1.4131	0.8693	−2.1244
PRO168	−0.9398	−0.0493	0	−0.0512	−0.9239	−0.9892	−0.1006	−1.0404	−0.9751	−1.9643

E_VDW_: van der Waals energy; E_EEL_: electrostatic energy; E_INT_: internal energy; G_POL_: the polar part of solvation free energy; E_SAS_: non-polar solvation component; E_MM_: gas phase Molecular Mechanics energy during binding; E_EELPOL_: electrostatic energy and the polar part of solvation free energy; E_VDWEELPOL_: van der Waals energy and electrostatic energy and the polar part of solvation free energy; G_GBSA_: Generalized Born Surface Area free energy; G_MM/GBSA_: Molecular Mechanics/Generalized Born Surface Area free energy.

**Table 5:** Energy decomposition of MM/GBSA free energy for the ZINC12530139. All energies are in kcal/mol.

Residue	E_VDW_	E_EEL_	E_INT_	G_POL_	E_SAS_	E_MM_	E_EELPOL_	E_VDWEELPOL_	G_GBSA_	G_MM/GBSA_
ASN142	−2.8483	−0.7788	0	0.1051	−1.774	−3.627	−0.6737	−3.522	−1.669	−5.296
GLN189	−1.2062	−2.8307	0	0.3067	−1.3396	−4.0368	−2.524	−3.7301	−1.0329	−5.0697
HIS164	−1.8092	−2.6969	0	0.5538	−0.9579	−4.5061	−2.1431	−3.9523	−0.4041	−4.9101
MET165	−2.4619	−0.3918	0	0.0996	−1.6995	−2.8537	−0.2922	−2.7541	−1.5999	−4.4536
GLY143	−1.6115	−1.0566	0	0.2761	−0.9806	−2.6681	−0.7805	−2.392	−0.7045	−3.3726
CYS145	−1.2837	−0.0759	0	−0.2514	−0.873	−1.3596	−0.3273	−1.611	−1.1244	−2.484
GLU166	−1.9215	2.6406	0	−1.892	−1.2823	0.7191	0.7486	−1.173	−3.1743	−2.4553
SER144	−1.0346	−0.6017	0	0.1506	−0.5429	−1.6363	−0.4511	−1.4857	−0.3923	−2.0286
LEU141	−1.0447	−0.5713	0	0.0816	−0.4866	−1.616	−0.4897	−1.5344	−0.405	−2.021
HIS41	−0.7741	−0.919	0	0.4292	−0.6046	−1.6931	−0.4898	−1.2639	−0.1755	−1.8685

E_VDW_: van der Waals energy; E_EEL_: electrostatic energy; E_INT_: internal energy; G_POL_: the polar part of solvation free energy; E_SAS_: non-polar solvation component; E_MM_: gas phase Molecular Mechanics energy during binding; E_EELPOL_: electrostatic energy and the polar part of solvation free energy; E_VDWEELPOL_: van der Waals energy and electrostatic energy and the polar part of solvation free energy; G_GBSA_: Generalized Born Surface Area free energy; G_MM/GBSA_: Molecular Mechanics/Generalized Born Surface Area free energy.

## Data Availability

Data is contained within the article.

## Supplementary Materials

The following supporting information can be downloaded at: https://www.mdpi.com/article/10.3390/molecules28030937/s1, Figure S1: The time course of root–mean–square deviations (RMSD) of top 1-5 ligands; Figure S2: The time course of root–mean–square deviations (RMSD) of top 6-10 ligands; Figure S3: The hydrogen bonding occupancy of ZINC15676170, ZINC09033700, and ZINC12530139; Figure S4: The time course of root–mean–square deviations (RMSD) plot.

## Sample Availability

Samples of the compounds are not available from the authors.
