# Supplementary material for: Discovery of Novel Chinese Medicine Compounds Targeting 3CL Protease by Virtual Screening and Molecular Dynamics Simulation
Source: Molecules. 2023 Jan 17;28(3):937. doi: 10.3390/molecules28030937 (PMC9921503; doi:10.3390/molecules28030937)

Supplemental Information

Discovery of Novel Chinese Medicines Compounds Targeting 3CL Protease by Virtual Screening and Molecular Dynamics Simulation

Jin Cheng[1#], Yixuan Hao[2#], Qin Shi[1#], Guanyu Hou[2], Yanan Wang[1], Yong Wang[1], Wen Xiao[1], Joseph Othman[2], Junnan Qi[2], Yuanqiang Wang[3*], Yan Chen[4*], Guanghua Yu[1*]

[1]School of Pharmacy, Jiangsu Vocational College of Medicine, Yancheng, 224005, China

[2]Department of Pharmaceutical Sciences and Computational Chemical Genomics Screening Center, School of Pharmacy; National Center of Excellence for Computational Drug Abuse Research; University of Pittsburgh, Pittsburgh, Pennsylvania 15261, United States.

[3]School of Pharmacy and Bioengineering, Chongqing University of Technology, Chongqing, China, 400054

[4]College of Pharmacology Sciences, Zhejiang University of Technology, Hangzhou, P.R. China, 310014

#These authors contributed equally

*To whom correspondence should be addressed: Guanghua Yu, School of Pharmacy, Jiangsu Vocational College of Medicine, Yancheng, 224005, China. Tel: +8613401772896; Email: 11303@jsmc.edu.cn; Yan Chen, College of Pharmacology Sciences, Zhejiang University of Technology, Hangzhou, P.R. China, 310014, Tel: +8657188813483; Email: chenyan2008@zjut.edu.cn; Yuanqiang Wang, School of Pharmacy and Bioengineering, Chongqing University of Technology, Chongqing, 400054, China. Tel: +862362563190; Email: wangyqnn@cqut.edu.cn.

Keywords: SARS-CoV-2, 3CL protease, virtual screening, traditional Chinese medicine, molecular dynamics simulations

Figure S1. The time course of root–mean–square deviations (RMSD) of top 1-5 ligands. (A) ZINC15676170, (B) ZINC15675325, (C) ZINC12529667, (D) ZINC13550544, (E) ZINC03838803.

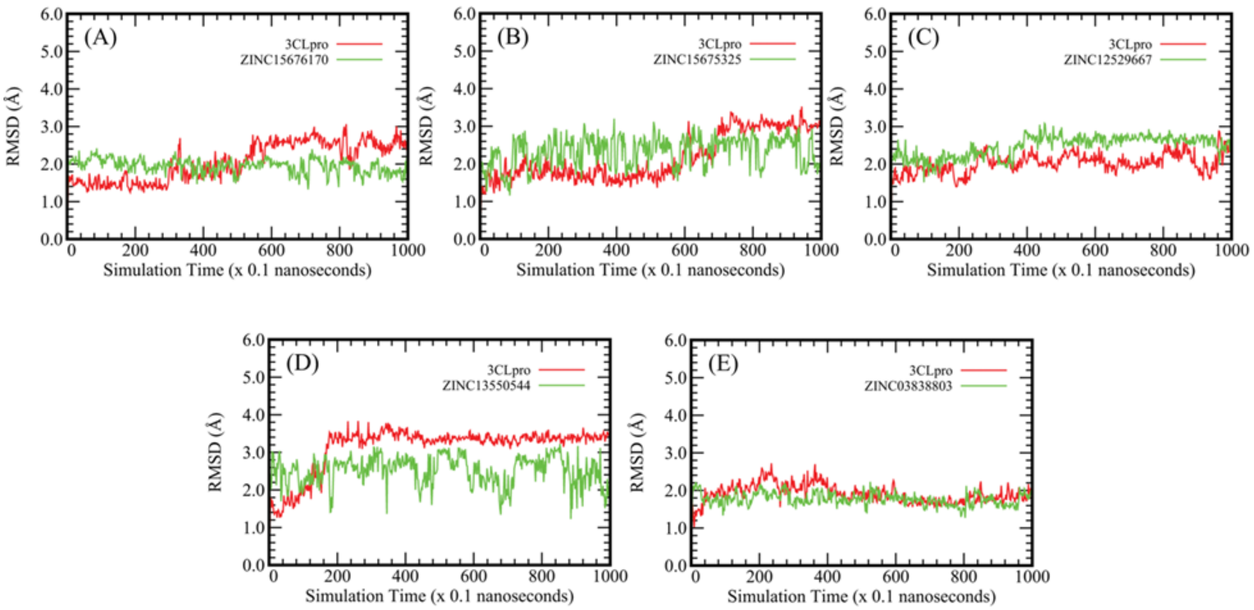

Figure S2. The time course of root–mean–square deviations (RMSD) of top 6-10 ligands. (A) ZINC12664661, (B) ZINC09033700, (C) ZINC12530139, (D) ZINC00198624, (E) ZINC08299537.

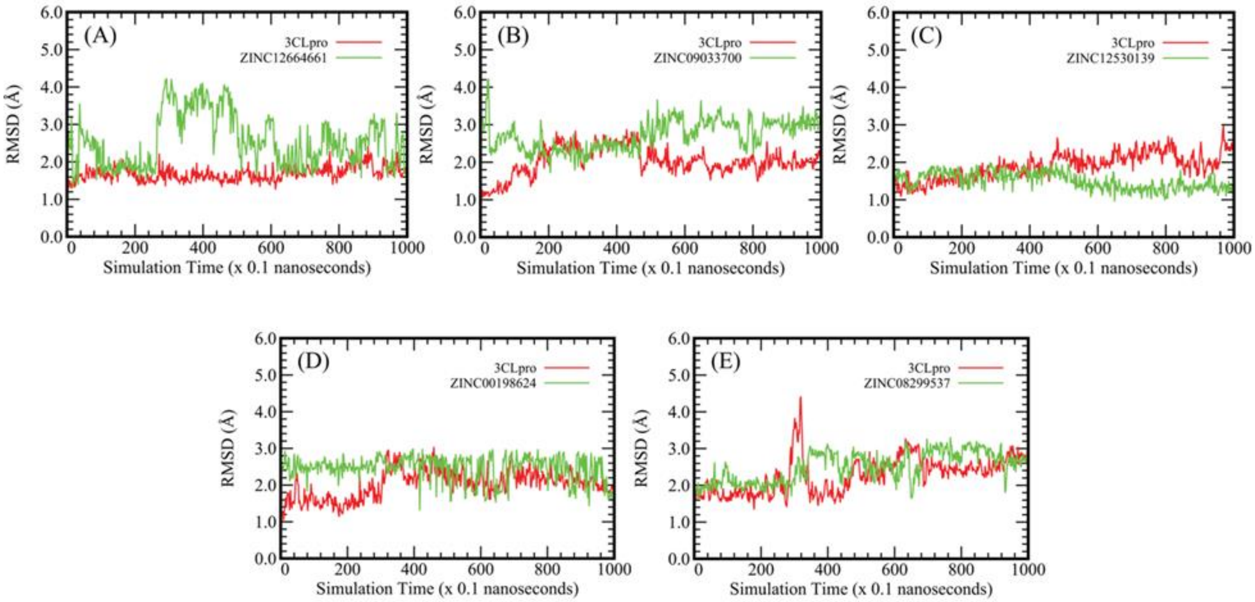

Figure S3. The hydrogen bonding occupancy of ZINC15676170, ZINC09033700, and ZINC12530139. In ZINC15676170, the hydrogen bonds of GLY143 and GLU166 accounted for 25.98% and 15.6% of the trajectory respectively. In ZINC09033700, the hydrogen bonds of GLU166 accounted for 21.5% of the trajectory. In ZINC12530139, the hydrogen bonds of GLY143 and SER144 accounted for 16.5% and 0.4% of the trajectory respectively.

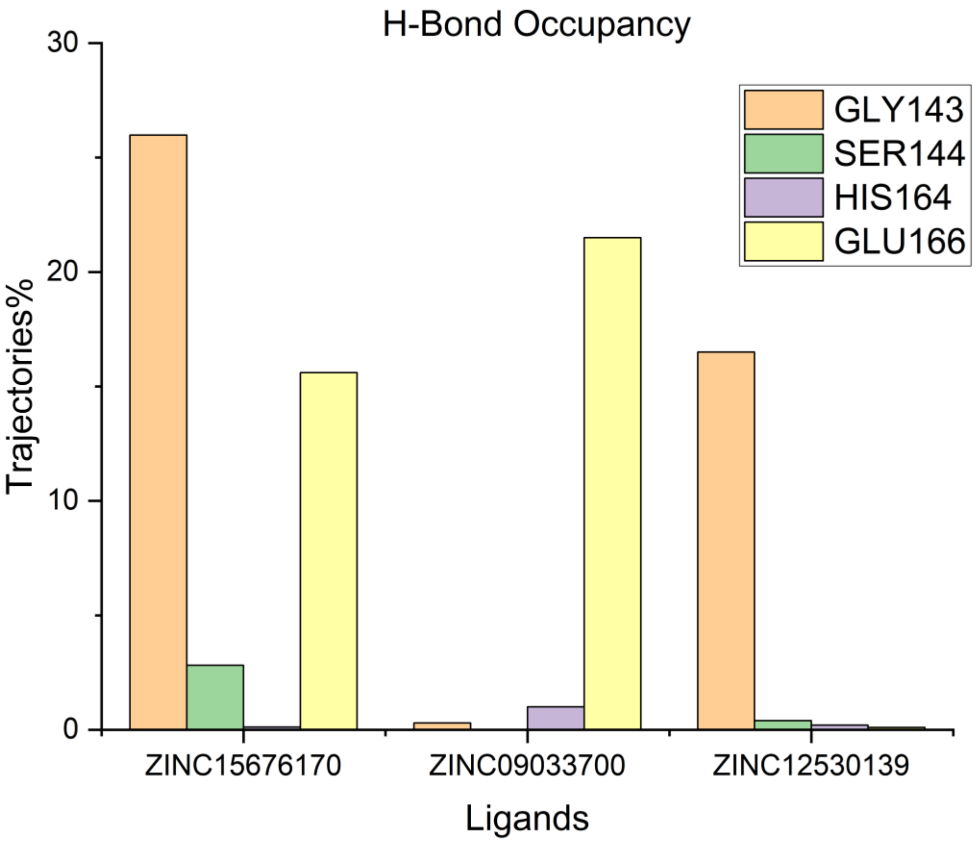

Figure S4. The time course of root–mean–square deviations (RMSD) plot of (A) ZINC15676170, (B) ZINC09033700, (C) ZINC12530139 (500ns).

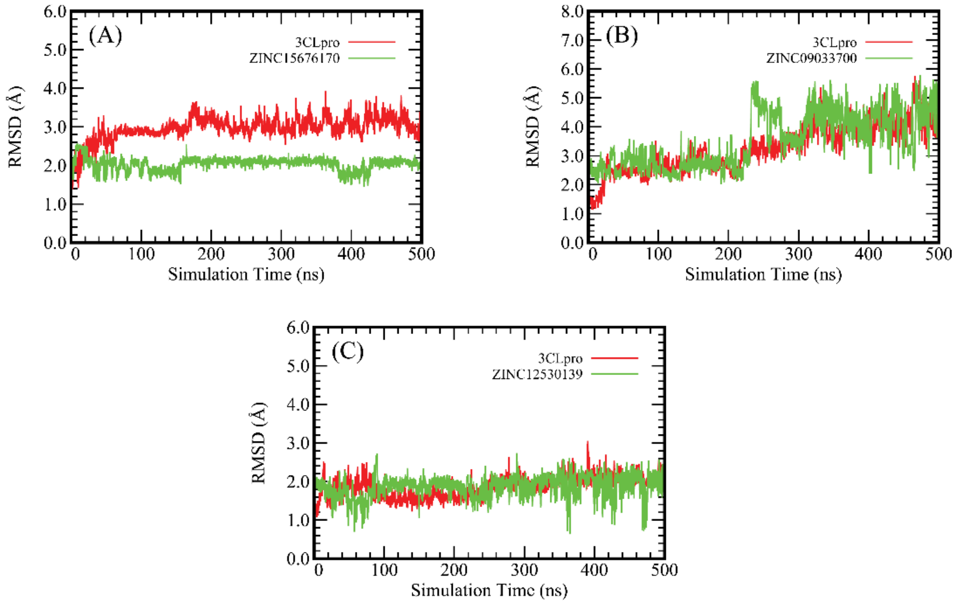

Supplement: Supplementary file 1 [file molecules-28-00937-s001.zip › molecules-2036381-supplementary.pdf]
